# Supplementary material for: A Comprehensive Assessment of Ultraviolet-Radiation-Induced Mutations in Flammulina filiformis Using Whole-Genome Resequencing
Source: J Fungi (Basel). 2024 Mar 20;10(3):228. doi: 10.3390/jof10030228 (PMC10971301; doi:10.3390/jof10030228)
Supplement: Supplementary file 1 [file jof-10-00228-s001.zip › Supplementary Material S8/KEGG annotation/out/64381550635650.os/KO/out_map/map04010.html]

KEGG PATHWAY: MAPK signaling pathway - Reference pathway


|  |  |
| --- | --- |
| **MAPK signaling pathway - Reference pathway** |  |

[
Pathway menu
| Organism menu
| Pathway entry
| Show description
| User data mapping
]

The mitogen-activated protein kinase (MAPK) cascade is a highly conserved module that is involved in various cellular functions, including cell proliferation, differentiation and migration. Mammals express at least four distinctly regulated groups of MAPKs, extracellular signal-related kinases (ERK)-1/2, Jun amino-terminal kinases (JNK1/2/3), p38 proteins (p38alpha/beta/gamma/delta) and ERK5, that are activated by specific MAPKKs: MEK1/2 for ERK1/2, MKK3/6 for the p38, MKK4/7 (JNKK1/2) for the JNKs, and MEK5 for ERK5. Each MAPKK, however, can be activated by more than one MAPKKK, increasing the complexity and diversity of MAPK signalling. Presumably each MAPKKK confers responsiveness to distinct stimuli. For example, activation of ERK1/2 by growth factors depends on the MAPKKK c-Raf, but other MAPKKKs may activate ERK1/2 in response to pro-inflammatory stimuli.

|  |  |
| --- | --- |
| Reference pathway | 100% |
